# Patient-Centered Outcomes After Minimally Invasive Internal Splinting Versus Open Achilles Tendon Repair: Comparable Clinically Meaningful Recovery at 12 Months

**DOI:** 10.3390/jcm15124570

**Published:** 2026-06-12

**Authors:** Recep Karasu, Mustafa Dinç

**Affiliations:** Department of Orthopedics and Traumatology, Bursa City Hospital, Bursa 16250, Turkey; drindianster@gmail.com

**Keywords:** Achilles tendon injuries, minimally invasive surgical procedures, minimal clinically important difference, patient satisfaction, patient reported outcome measures

## Abstract

**Background/Objectives**: Comparative studies evaluating minimally invasive surgery (MIS) and open repair for acute Achilles tendon rupture have predominantly relied on mean-based statistical comparisons, which may not adequately capture whether outcomes are clinically meaningful from the patient perspective. This study aimed to compare 12-month outcomes between MIS using the internal splinting technique and open repair, establish anchor-based minimal clinically important difference (MCID) thresholds, and compare patient-centered responder outcomes between techniques. **Methods**: This retrospective non-randomized comparative cohort study included 70 patients allocated to MIS (*n* = 35) or open repair *(n* = 35). Outcomes were assessed using VAS, AOFAS, ATRS, and Thermann score. Anchor-based MCID thresholds were determined via ROC curve analysis using the Global Rating of Change (GROC) scale as the external anchor. Patient Acceptable Symptom State (PASS) was assessed using a dichotomous anchor question. **Results**: Both groups demonstrated significant improvements across all outcome measures at 12 months (*p* < 0.001). No significant between-group differences were observed in mean functional scores, MCID achievement rates, PASS rates, or GROC-defined clinical success (*p* > 0.05 for all). AUC values ranged from 0.975 to 0.984. The MCID threshold for pain relief was identified as a VAS reduction > 4.8 points (AUC: 0.975, 95% CI: 0.906–0.998), while ROC-derived functional MCID thresholds were identified as an AOFAS increase >38 points (AUC: 0.984, 95% CI: 0.920–0.999), an ATRS increase >38 points (AUC: 0.984, 95% CI: 0.920–0.999), and a Thermann score increase >37 points (AUC: 0.984, 95% CI: 0.920–0.999). These thresholds should be considered exploratory and require validation in larger independent cohorts. MCID achievement rates were 42.9% for VAS in both groups, whereas MCID achievement for functional outcome measures (AOFAS, ATRS, and Thermann scores) was 62.9% in the MIS group and 57.1% in the open repair group. PASS-positive rates were 85.7% and GROC-defined clinical success 71.4% in both groups. Complication rates were low in both groups; however, the small number of events limits the strength of this conclusion, and larger studies are needed to evaluate potential between-group differences. **Conclusions**: Both techniques were associated with substantial clinically meaningful recovery at 12 months, and neither approach demonstrated a clear clinical advantage in patient-centered outcomes. The population-specific MCID thresholds derived in the present cohort may provide clinically interpretable benchmarks for future research, although external validation is required before broader application. Surgical decision-making may rely on surgeon expertise and patient factors rather than anticipated differences in patient-centered outcomes.

## 1. Introduction

Acute Achilles tendon rupture is a common lower extremity injury, with reported incidence rates ranging from approximately 15 to 40 per 100,000 person-years and a rising trend over recent decades, particularly among middle-aged individuals engaged in recreational sports [1,2]. Both operative and non-operative treatment strategies are available; however, surgical repair has been associated with significantly lower re-rupture rates and a faster return to pre-injury activity levels compared with conservative management [3,4]. Among surgical options, minimally invasive surgery (MIS) and open repair remain the most widely adopted techniques, each with distinct advantages and complication profiles [4,5].

Within the spectrum of MIS approaches, the internal splinting technique—originally described by Muezzinoglu et al. [6] and subsequently validated in a comparative study by Sarman et al. [7]—has emerged as a promising minimally invasive strategy for acute Achilles tendon repair. This technique bridges the rupture site using high-strength sutures passed percutaneously through small incisions, combining the biomechanical strength of open repair with the soft tissue preservation and paratenon integrity characteristic of percutaneous approaches [6,7]. By avoiding extensive longitudinal dissection, it aims to reduce iatrogenic vascular disruption, minimize wound-related complications, and facilitate more efficient functional recovery compared with conventional open techniques [7].

Unlike many percutaneous Achilles tendon repair techniques, which rely primarily on simple suture passage and may exhibit considerable variation in construct configuration [4,5], the internal splinting method creates a robust four-strand core repair construct comparable to open techniques while preserving paratenon integrity and avoiding extensive soft-tissue dissection [6,7]. Despite these theoretical advantages and encouraging early clinical results, evidence regarding the longer-term clinical effectiveness of this specific technique remains relatively limited [6,7].

Although numerous comparative studies have evaluated outcomes between MIS and open repair approaches, most have relied on traditional statistical comparisons of mean values derived from patient-reported outcome measures (PROMs) and clinician-administered scales, such as the Achilles Tendon Total Rupture Score (ATRS) [8], the American Orthopaedic Foot and Ankle Society (AOFAS) hindfoot score [9], the Thermann score [10], and the Visual Analogue Scale (VAS) for pain. While these analyses provide valuable group-level information, they do not directly address a key clinical question: whether the observed differences are meaningful from the patient’s perspective. As increasingly recognized in the orthopaedic literature, statistically significant differences may not necessarily translate into clinically relevant benefit for individual patients [11,12,13].

To address this limitation, the concepts of minimal clinically important difference (MCID) and patient acceptable symptom state (PASS) have gained increasing attention in orthopaedic research [12,14,15]. The MCID, first described by Jaeschke et al. [14], represents the smallest change in a patient-reported outcome measure that patients perceive as beneficial. In contrast, the PASS, originally established by Tubach et al. [15], reflects the absolute symptom state beyond which patients consider themselves well or satisfied with their current condition. The Global Rating of Change (GROC) scale serves a different purpose, capturing the patient’s overall perception of improvement since treatment and frequently acting as an external anchor for MCID determination. Thus, MCID quantifies meaningful change, PASS reflects an acceptable current state, and GROC measures perceived improvement over time. Together, these complementary concepts provide a more comprehensive and patient-centered assessment of treatment effectiveness than conventional mean-based analyses alone.

However, despite their growing importance, several studies have reported MCID and PASS thresholds and related patient-centered outcome analyses in patients with Achilles tendon rupture [16,17,18]. Nevertheless, reported thresholds vary considerably across studies because of differences in patient populations, treatment strategies, outcome instruments, and methodological approaches. Furthermore, most available evidence has focused on mixed operative and non-operative cohorts or on the validation of individual outcome measures, whereas patient-centered comparisons between specific surgical techniques remain limited. Consequently, the clinical applicability of existing thresholds to surgically treated Achilles tendon rupture populations, particularly when comparing minimally invasive and open repair techniques, remains insufficiently characterized [17,18,19].

Furthermore, the comparative effectiveness of the internal splinting MIS technique versus open repair has been evaluated only to a limited extent using patient-centered outcome measures such as MCID and PASS. It remains unclear whether the potential early advantages of MIS translate into clinically meaningful patient-perceived benefits at 12 months—a gap that limits the ability to interpret outcomes in a clinically relevant manner and constrains evidence-based shared decision-making.

Therefore, the present study was designed with three primary objectives: (1) to compare 12-month clinical outcomes, including pain and functional recovery, between MIS using the internal splinting technique and open Achilles tendon repair; (2) to determine anchor-based MCID thresholds for VAS, ATRS, AOFAS, and the Thermann score using receiver operating characteristic (ROC) analysis with the Global Rating of Change (GROC) scale as the external anchor, and to assess PASS achievement rates using a dichotomous acceptable symptom state question; and (3) to compare MCID achievement rates and PASS rates between the two techniques, thereby providing a patient-centered interpretation of treatment effectiveness.

We hypothesized that minimally invasive repair using the internal splinting technique would demonstrate no clinically meaningful differences in patient-centered outcomes compared with open repair at 12 months, while potentially reducing soft-tissue morbidity. We further hypothesized that MCID- and PASS-based analyses would provide additional clinically relevant information beyond conventional mean-based outcome comparisons.

## 2. Materials and Methods

### 2.1. Study Design and Ethical Approval

This was a non-randomized, retrospective comparative cohort study conducted at Bursa City Hospital, Bursa, Turkey, between January 2021 and December 2024. The study protocol was approved by the Bursa City Hospital Scientific Research Ethics Committee (approval no. 2025-16/8, approved 20 August 2025) and was conducted in accordance with the principles of the Declaration of Helsinki. Given the retrospective design of the study, the requirement for individual informed consent was waived by the institutional review board.

### 2.2. Participants

Patients were identified through a retrospective review of institutional surgical records between January 2021 and December 2024. A total of 90 patients with acute unilateral Achilles tendon rupture were screened for eligibility. Twenty patients were excluded: 9 due to chronic tendinopathy or prior Achilles tendon surgery, 6 due to prior ipsilateral foot and ankle surgery, and 5 due to incomplete follow-up or unavailable outcome data. Of these 5 patients, 3 were lost to follow-up (2 relocated to another city and 1 failed to attend the 12-month follow-up visit despite repeated contact attempts), while 2 had incomplete outcome data that could not be reliably retrieved from the medical records. The remaining 70 patients met all inclusion criteria and were included in the final analysis. Diagnosis was established based on clinical examination and confirmed by magnetic resonance imaging (MRI) and/or ultrasonography.

Inclusion criteria were: (1) complete Achilles tendon rupture within 10 days of injury, (2) suitability for surgical repair, and (3) ability to comply with the postoperative rehabilitation and follow-up protocol. Exclusion criteria included: (1) recurrent Achilles tendon rupture, (2) chronic tendinopathy or prior Achilles tendon surgery, (3) prior ipsilateral foot and ankle surgery, (4) concomitant lower extremity fractures, (5) peripheral neuropathy or systemic inflammatory disease, and (6) incomplete follow-up or unavailable outcome data.

The 70 included patients were treated with either the minimally invasive surgery (MIS) technique (*n* = 35) or open repair (*n* = 35) based on patient preference and surgeon consensus. Treatment allocation was not randomized. Following confirmation of Achilles tendon rupture, patients were informed about both surgical options, including differences in incision size, soft-tissue dissection, potential wound-related complications, and postoperative rehabilitation. The final treatment decision was made jointly by the patient and the treating surgeon after discussion of the risks and benefits of each technique. No predefined demographic, radiological, or injury-related criteria were used to preferentially assign patients to either treatment group. Consequently, treatment selection was influenced by patient preferences (e.g., cosmetic concerns and perceived risk of wound complications) and the surgeon’s assessment of soft-tissue condition and rupture configuration. Baseline demographic and clinical characteristics were compared between groups prior to outcome analysis.

### 2.3. Surgical Techniques

All procedures were performed by a single fellowship-trained foot and ankle surgeon with extensive experience in both techniques. A standardized postoperative rehabilitation protocol was applied to all patients.

#### 2.3.1. Minimally Invasive Surgery (MIS)—Internal Splinting Method

The MIS technique was performed using a modified internal splinting method, as originally described by Muezzinoglu et al. [6] and subsequently validated by Sarman et al. [7] Two small medial incisions (approximately 1–2 cm) were made proximal and distal to the rupture site, and the paratenon was carefully incised and preserved as much as possible. Using a curved tendon needle, No. 2 non-absorbable braided sutures (FiberWire; Arthrex, Naples, FL, USA) were passed through both tendon stumps in a locking Krackow configuration, bridging the rupture site and combining the biomechanical advantages of open repair with the soft tissue preservation of percutaneous techniques. Sutures were tied securely with the ankle in equinus position, and the knots were buried beneath the paratenon. Skin closure was achieved using subcuticular sutures.

#### 2.3.2. Open Repair Method

Open repair was performed through a longitudinal posteromedial incision (approximately 6–8 cm) centered over the rupture site. The paratenon was incised, and minimal debridement of tendon ends was performed. Tendon repair was carried out using a modified Kessler or Bunnell technique with non-absorbable sutures. The paratenon was re-approximated, and the skin was closed in layers.

### 2.4. Postoperative Rehabilitation

A standardized rehabilitation protocol was applied uniformly to both groups. Patients were immobilized in a below-knee cast with the ankle in equinus position for the first 2 weeks. Thereafter, a removable walking boot with heel lifts was used for an additional 4 weeks, with progressive weight-bearing as tolerated. Passive ankle dorsiflexion and active plantarflexion exercises were initiated at 2 weeks postoperatively. Strengthening exercises were introduced at 6 weeks. Return to sports activities was permitted between 4 and 6 months based on clinical assessment.

### 2.5. Outcome Measures and Follow-Up

Outcome data were extracted from medical records at preoperative baseline and at the 12-month postoperative follow-up. The following clinical outcome measures were recorded:

Pain was assessed using the Visual Analogue Scale (VAS; 0–10, lower scores indicate less pain).

Patient-reported function was evaluated using the Achilles Tendon Total Rupture Score (ATRS; 0–100, higher scores indicate better function), the only PROM specifically developed and validated for Achilles tendon rupture [8].

Clinician-influenced functional assessment was performed using the American Orthopaedic Foot and Ankle Society (AOFAS) hindfoot score (0–100), as described by Kitaoka et al. [9].

Additional functional assessment was performed using the Thermann score (0–100, higher scores indicate better outcomes), a composite clinician-administered instrument evaluating pain, functional capacity, and activity level following Achilles tendon repair, as originally described by Thermann et al. [10].

### 2.6. Anchor Measures

At the 12-month follow-up, anchor-based assessments were obtained to evaluate clinically meaningful improvement and patient-acceptable outcomes. The Global Rating of Change (GROC) scale was administered using a 5-point Likert scale ranging from 1 (“much worse”), 2 (“worse”), 3 (“unchanged”), 4 (“better”), to 5 (“much better”), as originally described by Jaeschke et al. [14]. The minimal clinically important difference (MCID) is defined as the smallest change in a patient-reported outcome measure that patients perceive as beneficial and that may justify a change in patient management. Anchor-based methods, which compare changes in outcome scores with an external criterion such as the GROC scale, are recommended as the primary approach for estimating MCID thresholds because they directly reflect patients’ perspectives [20,21,22]. For MCID determination, patients with GROC scores of 4 (“better”) or 5 (“much better”) were classified as having achieved clinically meaningful improvement and constituted the positive anchor group, whereas patients with scores of 1 to 3 (“much worse,” “worse,” or “unchanged”) constituted the reference group for ROC analysis. This classification is consistent with established anchor-based methodology, in which patients reporting that they were “better” or “much better” are considered responders for MCID estimation [18,22,23].

The Patient Acceptable Symptom State (PASS) was assessed at final follow-up using a single dichotomous question, as described by Tubach et al. [15]: “Considering all the ways your condition affects you, do you consider your current state satisfactory?” Patients responding “Yes” were classified as having achieved an acceptable symptom state. To minimize potential recall and ordering bias, the PASS question was administered before the Global Rating of Change (GROC) scale. Patients were first asked whether they considered their current symptom state acceptable and were subsequently asked to rate their perceived change since treatment using the GROC scale. Both GROC and PASS assessments were obtained by the operating surgeon during routine 12-month postoperative outpatient follow-up visits and documented in the patients’ clinical records as part of standard postoperative evaluation. These data were subsequently extracted retrospectively from the medical records for the purposes of the present study.

Baseline demographic and clinical characteristics—including age, sex, body mass index (BMI), injured side, dominant limb involvement, mechanism of injury, rupture level, time to surgery, smoking status, and diabetes mellitus—were retrospectively retrieved from institutional medical records.

### 2.7. Statistical Analysis

No formal a priori sample size calculation was performed, given the retrospective design of the study. The sample size of 35 patients per group was considered adequate and consistent with previously published comparative studies evaluating minimally invasive and open Achilles tendon repair techniques [4,7,24]. Consequently, the absence of a formal sample size calculation limits the interpretation of non-significant between-group findings, particularly for responder outcomes (MCID and PASS achievement rates) and complication rates, as the study may have been underpowered to detect small but potentially clinically meaningful differences.

The normality of data distribution was assessed using the Shapiro–Wilk test. Continuous variables were expressed as mean ± standard deviation (SD) for normally distributed data and as median (minimum–maximum) for non-normally distributed variables. Between-group comparisons (MIS vs. OPEN) were performed using the Independent Samples *t*-test for normally distributed variables and the Mann–Whitney U test for non-normally distributed variables. Within-group changes from preoperative baseline to final follow-up were analyzed using the Paired Samples *t*-test or Wilcoxon Signed-Rank test, as appropriate. Categorical variables were presented as frequencies and percentages and compared using the Pearson chi-square test or Fisher’s exact test when appropriate. Postoperative complication rates were compared between groups using Fisher’s exact test.

The Minimal Clinically Important Difference (MCID) for each clinical outcome measure was determined using an anchor-based approach through Receiver Operating Characteristic (ROC) curve analysis. The GROC scale was used as the external anchor variable, as described above. Prior to MCID estimation, the suitability of the GROC scale as an external anchor was assessed by examining its association with each outcome change score. Spearman correlation coefficients between GROC ratings and change scores were strong for all outcome measures (ΔVAS: r = −0.829; ΔAOFAS: r = 0.847; ΔATRS: r = 0.847; ΔThermann: r = 0.847; all *p* < 0.001), exceeding the recommended threshold of r > 0.30–0.35 for anchor validity in MCID studies [21,22,25,26]. The optimal MCID cut-off values were identified using the Youden Index. The discriminative ability of each clinical score was quantified by calculating the Area Under the ROC Curve (AUC) with corresponding 95% confidence intervals (CI). Pairwise comparisons of ROC curves were performed using the DeLong method. Because the MCID thresholds were derived and subsequently evaluated within the same study cohort, they should be considered exploratory and sample-specific. External validation in larger independent cohorts is required before broader clinical application.

PASS achievement rates were compared between groups using the Pearson chi-square test. All statistical analyses were conducted using IBM SPSS Statistics for Windows, version 25.0 (IBM Corp., Armonk, NY, USA). A two-sided *p*-value < 0.05 was considered statistically significant.

## 3. Results

### 3.1. Baseline Demographic and Clinical Characteristics

A total of 70 patients were included in the study, with 35 patients included in the minimally invasive surgery (MIS) group and 35 in the open repair group. The two cohorts were well-matched across all baseline demographic and clinical variables, with no statistically significant differences detected between groups (*p* > 0.05 for all; Table 1). Mean age was 42.26 ± 8.30 years in the MIS group and 42.74 ± 8.39 years in the Open Repair group (*p* = 0.808), and mean body mass index was comparable between groups (27.61 ± 2.19 vs. 27.62 ± 2.18 kg/m^2^; *p* = 0.987). Sex distribution, rates of smoking and diabetes mellitus, injury laterality, dominant limb involvement, and mechanism of injury were comparable between the two cohorts (*p* = 1.000 for all). Preoperative rupture level (4.71 ± 0.83 vs. 4.76 ± 0.84 cm; *p* = 0.831) and median time to surgery [5 (range: 3–8) days in both groups; *p* = 1.000] also did not differ significantly between groups. In addition, standardized mean differences (SMDs) were uniformly small (all SMDs < 0.10), indicating negligible baseline imbalance between the MIS and open repair groups.

### 3.2. Functional Outcomes: Within-Group Analysis

Both surgical groups demonstrated statistically significant improvements across all clinical outcome measures from preoperative baseline to the 12-month follow-up (Table 2). In the MIS group, the mean VAS score decreased from 8.05 ± 0.29 preoperatively to 3.16 ± 0.59 at final follow-up (*p* < 0.001), and mean AOFAS, ATRS, and Thermann scores improved significantly from preoperative baseline values to 83.57 ± 3.73, 81.57 ± 3.73, and 82.57 ± 3.73, respectively (*p* < 0.001 for all). In the Open Repair group, VAS scores declined from 8.06 ± 0.29 to 3.23 ± 0.56, with comparable significant improvements observed across all functional outcome measures (Table 2; *p* < 0.001 for all).

### 3.3. Functional Outcomes: Between-Group Comparison of Clinical Improvement

The magnitude of improvement from preoperative baseline to final follow-up, expressed as delta scores, was comparable between the MIS and Open Repair groups for all clinical outcome measures (*p* > 0.05 for all; Table 3). The median delta VAS score was −4.80 in both groups [MIS: −4.80 (range: −6.00 to −4.30); Open Repair: −4.80 (range: −5.40 to −4.20); *p* = 0.731]. Similarly, median delta AOFAS and ATRSs were 39 points in both cohorts [MIS: 39 (35–44); Open Repair: 39 (34–41); *p* = 0.559], while median delta Thermann scores were 38 points in both groups [MIS: 38 (34–43); Open Repair: 38 (33–40); *p* = 0.559]. These findings indicate that both surgical techniques provided comparable levels of pain relief and functional recovery during the follow-up period.

### 3.4. MCID Determination: ROC Curve Analysis

The Minimal Clinically Important Difference (MCID) for each clinical outcome measure was established through anchor-based ROC curve analysis using the GROC scale as the external anchor (positive group: GROC 4–5, *n* = 50; reference group: GROC 1–3, *n* = 20). All four delta scores demonstrated high discriminative capacity, with AUC values ranging from 0.975 to 0.984 (*p* < 0.001 for all; Table 4).

Based on the Youden Index, the MCID threshold for pain relief was identified as a VAS reduction of >4.8 points (AUC: 0.975, 95% CI: 0.906–0.998; sensitivity: 88.0%; specificity: 95.0%; PPV: 97.8%; NPV: 76.0%). For functional outcome measures, MCID thresholds were established as increases of >38 points for both AOFAS and ATRSs (AUC: 0.984, 95% CI: 0.920–0.999; sensitivity: 84.0%; specificity: 100.0%; PPV: 100.0%; NPV: 71.4%) and >37 points for the Thermann score (AUC: 0.984, 95% CI: 0.920–0.999; sensitivity: 84.0%; specificity: 100.0%; PPV: 100.0%; NPV: 71.4%).

**Figure 1:**
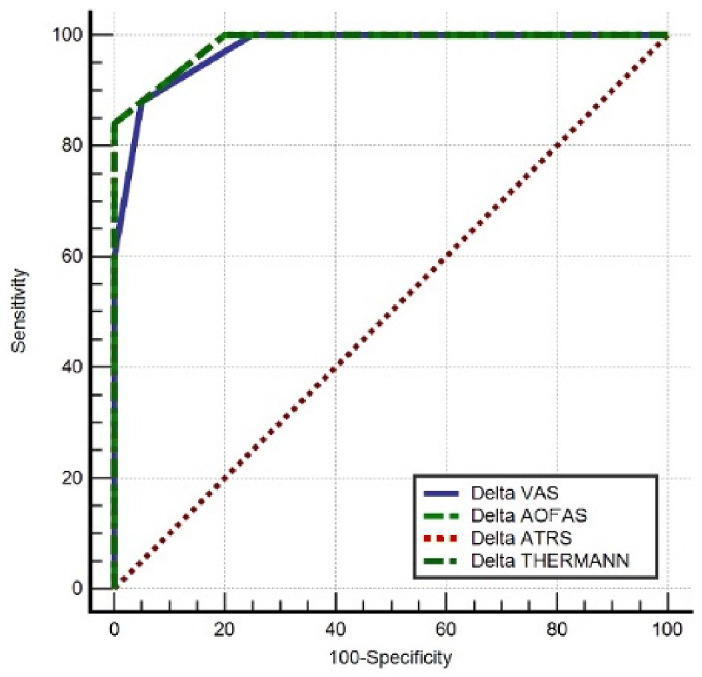
Combined Receiver Operating Characteristic (ROC) curves of clinical improvement scores. The graph illustrates the discriminative capacity of the delta (change) scores for the Visual Analogue Scale (VAS), American Orthopaedic Foot and Ankle Society (AOFAS) score, Achilles Tendon Total Rupture Score (ATRS), and Thermann score in identifying patients who achieved a minimal clinically important difference. Area Under the Curve (AUC) values for all parameters ranged from 0.975 to 0.984 (*p* < 0.001), indicating excellent discriminative ability. Pairwise comparisons of the curves using the DeLong method revealed no statistically significant differences in AUC values among the four clinical scales (*p* > 0.05 for all comparisons). The diagonal reference line represents a non-discriminatory test (AUC = 0.5).

Pairwise comparisons of ROC curves using the DeLong method revealed no statistically significant differences in AUC values among the four clinical scales (*p* > 0.05 for all comparisons), indicating comparable discriminative performance across pain and functional outcome measures in identifying patients who achieved clinically meaningful improvement (Figure 1).

### 3.5. MCID Achievement Rates

The proportion of patients meeting the MCID threshold at final follow-up was comparable between the MIS and Open Repair groups across all evaluated outcome measures (Table 5). For pain relief, the VAS MCID threshold was achieved by 42.9% of patients in both groups (*p* = 1.000). With respect to functional recovery, MCID achievement rates for AOFAS, ATRS, and Thermann scores were numerically higher in the MIS group (62.9%) than in the Open Repair group (57.1%); however, these differences did not reach statistical significance (*p* = 0.626 for all). No significant between-group differences were observed in the proportion of patients achieving clinically meaningful improvement for any outcome measure.

### 3.6. Patient Acceptable Symptom State and Clinical Success

At the 12-month follow-up, patient-reported symptom acceptability and overall clinical success were similar between the two groups (Table 6). A total of 30 patients in each group (85.7%) reported their current symptom state as acceptable based on the PASS question. According to GROC-based clinical success classification, 25 patients (71.4%) in both the MIS and Open repair groups achieved a successful outcome. No statistically significant differences were detected between the two techniques regarding PASS rates or GROC-defined clinical success (*p* = 1.000 for both comparisons).

### 3.7. Postoperative Complications

Postoperative complications were infrequent in both groups and no statistically significant between-group differences were detected for any individual complication (*p* > 0.05 for all; Table 7). In the MIS group, one patient (2.9%) developed a superficial wound infection and one patient (2.9%) sustained a re-rupture. In the Open Repair group, two patients (5.7%) developed superficial wound infections, two patients (5.7%) sustained re-ruptures, and two patients (5.7%) experienced wound necrosis. No cases of deep infection or sural nerve injury were recorded in either group.

## 4. Discussion

The present study indicates that both MIS and open repair provide substantial and clinically meaningful recovery following acute Achilles tendon rupture. Within-group analyses showed marked improvements in pain relief and functional outcomes across all evaluated clinical scales, including VAS, AOFAS, ATRS, and Thermann scores, at 12 months, suggesting that both techniques represent highly effective surgical treatment strategies when assessed independently.

More importantly, however, the principal finding of this study extends beyond the simple absence of statistically significant between-group differences. Although conventional comparative analyses demonstrated similar magnitudes of improvement between MIS and open repair for all evaluated outcome measures, the incorporation of patient-centered outcome methodology provided a more comprehensive understanding of clinical effectiveness from the patient perspective.

When recovery was evaluated using anchor-based MCID analysis, both techniques demonstrated similar rates of clinically meaningful improvement across all evaluated pain and functional outcome measures. The likelihood of achieving an improvement that patients themselves perceive as meaningful was essentially identical between groups. Likewise, PASS and GROC analyses—which directly reflect the patient’s subjective perception of recovery, symptom acceptability, and overall treatment success—yielded very similar findings for both surgical approaches. Neither the proportion of patients reaching an acceptable symptom state nor the proportion reporting successful clinical recovery differed between MIS and open repair.

The consistency of these findings across multiple complementary analytical frameworks is particularly compelling. Statistical non-significance alone does not necessarily imply clinical similarity, as similar mean improvements may still mask meaningful differences in patient-perceived recovery. However, in the present study, highly similar outcomes were observed not only through traditional statistical comparisons, but also through responder analyses, anchor-based clinical thresholds, and patient satisfaction metrics. This convergence suggests that MIS and open Achilles tendon repair produce similarly favorable clinical outcomes within the limitations of the present study design.

Accordingly, the findings indicate that both techniques were associated with substantial improvements in pain, function, and patient-reported recovery. Both approaches effectively reduced pain, restored ankle function, improved Achilles tendon-specific recovery, achieved high rates of clinically meaningful improvement, and generated high levels of patient satisfaction and perceived treatment success. Within the limits of the present follow-up period and evaluated outcomes, neither surgical technique demonstrated a clear clinical advantage over the other.

The absence of statistically significant differences between groups should not be interpreted as evidence of equivalence, as the present study was not designed as a non-inferiority or equivalence trial. Rather, the findings indicate that no clear clinical advantage of one technique over the other was detected within the evaluated outcomes and follow-up period.

These findings are broadly consistent with the existing literature demonstrating that both minimally invasive surgery (MIS) and open repair provide effective treatment for acute Achilles tendon rupture. However, the present study extends the current evidence by integrating responder-based and patient-centered outcome assessments (MCID, PASS, GROC) to evaluate clinical effectiveness beyond conventional mean comparisons.

Several previous studies have reported potential advantages of MIS, particularly regarding reduced soft-tissue disruption, lower wound complication rates, and improved early postoperative recovery. Cao et al. [27] demonstrated reduced operative time and lower early postoperative pain with MIS, while noting that functional scores converged between groups at longer follow-up. Similarly, the meta-analysis by Attia et al. [4] confirmed lower wound complication rates with MIS but concluded that long-term functional outcomes remain largely comparable between techniques. Consistent with these reports, the present study also observed a numerically lower overall complication rate in the MIS group, driven primarily by the absence of wound necrosis; however, this difference did not reach statistical significance. Li et al. [24] reported favorable early functional recovery with mini-open techniques, again emphasizing comparable longer-term results. Sarman et al. [7] demonstrated superior early functional outcomes and faster return to activity with semi-invasive internal splinting, yet long-term comparability persisted.

The present study supports and refines this concept of 12-month comparability. In contrast to many previous investigations that relied exclusively on mean-based comparisons, the current study additionally incorporated anchor-based MCID, PASS, and GROC analyses. Importantly, the absence of statistically significant between-group differences was consistently observed across all analytical frameworks—not only for mean score improvements, but also for the proportions of patients achieving clinically meaningful thresholds, acceptable symptom states, and successful global recovery. Thus, the outcomes reported in the previous literature appear to extend to the individual patient’s perception of meaningful recovery and treatment satisfaction, although definitive conclusions about comparability require further research.

The present findings therefore provide a more nuanced interpretation of clinical comparability. While some earlier studies have suggested potential early advantages of MIS, the current results indicate that these advantages do not translate into superior patient-perceived clinical meaningfulness or satisfaction at 12 months. This observation aligns with the convergence patterns reported by Cao et al. [27] and the meta-analytic conclusions of Attia et al. [4], while adding the critical insight that even responder-based thresholds (MCID, PASS, GROC) did not demonstrate clinically meaningful superiority of either technique. This convergence across multiple analytical approaches supports the interpretation that MIS and open repair produce similarly favorable patient-centered outcomes, without implying statistical equivalence.

The MCID thresholds and PASS rates of the current study contribute important context-specific benchmarks to the literature. Thresholds for clinically meaningful improvement after Achilles tendon rupture remain heterogeneous across studies, partly due to differences in patient populations, treatment strategies, and methodological approaches. Costa et al. [28] established an ATRS MCID of 8 points in a conservatively managed cohort, a value subsequently applied by Le J et al. [19] in their Bayesian re-analysis of the PATH-2 trial—substantially lower than the threshold of >38 points identified in the present surgically treated population. Dams et al. [18] reported minimally important change (MIC) values of 13.5 and 28.5 for the ATRS in a mixed population including both surgical and non-surgical treatments. In contrast, the present surgically treated cohort yielded substantially higher ATRS MCID thresholds (>38 points). This difference likely reflects both the greater magnitude of recovery achievable with surgical intervention and the higher functional expectations of surgically managed patients.

For PASS, Larsson et al. [17] and Cramer et al. [16] reported ATRS-based threshold values of approximately 75 and 57 points, respectively, in mixed populations. In the present study, PASS was operationalized as a patient-reported achievement rate using a dichotomous anchor question rather than a score-based threshold, as the limited number of patients reporting an unacceptable symptom state precluded reliable ROC-derived estimation. Nevertheless, the high PASS achievement rate observed in both groups (85.7%), together with median postoperative ATRSs exceeding the thresholds reported in these mixed cohorts, suggests that postoperative recovery in the present surgically treated population generally exceeded the acceptability benchmarks previously reported in heterogeneous or non-operative populations. These observations further emphasize the importance of context-specific outcome interpretation rather than direct extrapolation from heterogeneous patient populations with differing treatment strategies.

Regarding outcome instruments, the present findings support the complementary use of both general functional and tendon-specific assessment tools. The ATRS, as a fully patient-reported and Achilles tendon-specific instrument, demonstrated strong responsiveness and excellent discriminative performance in MCID analyses. The AOFAS and Thermann scores showed highly consistent patterns with ATRS findings, reinforcing internal consistency across different assessment modalities. However, because AOFAS includes clinician-administered components, future studies may continue to prioritize ATRS as the primary patient-reported outcome measure in this population. To our knowledge, this study provides the first anchor-based MCID thresholds for AOFAS in this population, along with PASS achievement rates, offering a useful reference for future comparative research.

Collectively, the present study suggests that both MIS and open Achilles tendon repair yield similarly favorable and clinically meaningful recovery at 12 months, not only in conventional mean-based analyses but also across responder-based assessments (MCID, PASS, and GROC). However, the non-randomized design and lack of equivalence testing preclude definitive conclusions regarding true clinical comparability between the two techniques. By demonstrating that early technical advantages of MIS do not translate into superior patient-perceived clinical meaningfulness, the study refines the interpretation of treatment comparability and provides clinically relevant benchmark data to guide future outcome interpretation and evidence-based postoperative assessment.

Beyond clinical comparisons, the theoretical biological advantages of MIS warrant consideration within the context of the present findings. The Achilles tendon derives a substantial portion of its extrinsic vascular supply from the paratenon, which serves as an important conduit for peritendinous perfusion [29,30]. Experimental studies have shown that surgical dissection reduces local tendon blood flow, thereby compromising the biological environment necessary for optimal healing [29]. In open repair, wider longitudinal exposure may impair paratenon continuity and increase soft tissue trauma, whereas minimally invasive approaches aim to preserve peritendinous structures and reduce iatrogenic tissue disruption [31]. These theoretical biological advantages have been proposed as potential mechanisms underlying the lower wound complication rates and improved early postoperative recovery reported in MIS studies [4,31]. In the present cohort, wound necrosis was observed exclusively in the Open Repair group and the overall complication rate was numerically lower in the MIS group, a pattern that may be consistent with the paratenon-preserving biological rationale proposed for minimally invasive techniques.

Similarly, the extent of surgical dissection may influence postoperative inflammatory responses and peritendinous adhesion formation [32]. Greater soft tissue disruption has been associated with increased local inflammatory signaling and fibroproliferative activity, potentially contributing to impaired tendon gliding and restricted ankle mobility [32]. By minimizing surgical exposure and preserving the gliding interface, MIS may reduce adhesion formation and facilitate earlier functional recovery [5,33].

However, the present findings suggest that these potential biological and biomechanical advantages do not necessarily translate into superior patient-perceived recovery at 12 months. Despite the theoretical benefits of reduced tissue disruption and preserved vascularity, no significant differences were observed between MIS and open repair regarding mean functional improvement, MCID achievement, PASS rates, or GROC-defined clinical success. These findings indicate that although MIS may confer certain biological or perioperative advantages, both techniques ultimately achieve similarly favorable levels of clinically meaningful recovery when combined with standardized rehabilitation protocols, though causal inferences are limited by the study design. However, these proposed mechanisms remain speculative within the context of the present study, as vascularity, tendon morphology, inflammatory activity, and adhesion formation were not directly assessed. Therefore, the biological explanations discussed above should be interpreted as theoretical mechanisms supported by previous literature rather than findings directly demonstrated by the present data.

The absence of clinically meaningful superiority in the present study may reflect several factors. Modern open repair techniques increasingly emphasize meticulous soft tissue handling and preservation of paratenon integrity, potentially reducing historical differences between approaches. In addition, postoperative rehabilitation likely plays a major role in determining functional recovery, possibly attenuating the influence of modest surgical biological differences over time. Consequently, while MIS remains biologically attractive from a tissue-preservation perspective, the current findings suggest that these mechanistic advantages may not be sufficient to produce detectable differences in patient-centered outcomes at 12 months.

Despite its retrospective design, this study incorporates several methodological features that enhance the validity and clinical relevance of its findings. All procedures were performed by a single fellowship-trained foot and ankle surgeon using standardized surgical techniques, minimizing inter-surgeon variability. In addition, a uniform postoperative rehabilitation protocol was applied across both groups, thereby reducing rehabilitation-related confounding and allowing a more isolated evaluation of surgical technique. Baseline demographic and clinical characteristics were systematically compared and found to be comparable between groups, partially mitigating allocation bias.

A major methodological strength of the present study is the integration of anchor-based MCID threshold analyses using ROC methodology, alongside PASS achievement rate assessment and GROC-defined clinical success classification, providing a patient-centered and clinically interpretable framework beyond conventional mean-based comparisons. Rather than relying solely on statistical significance testing, the study evaluated whether postoperative improvements were clinically meaningful from the patient perspective. This multidimensional analytical approach supports the interpretation of treatment comparability by demonstrating consistent findings across mean-based analyses, responder thresholds, patient-acceptable symptom states, and global recovery assessments. The use of multiple validated outcome instruments encompassing pain, functional recovery, and patient-perceived satisfaction further enhances the robustness and clinical relevance of the findings.

Several limitations must nonetheless be acknowledged. The retrospective design remains the most fundamental limitation, as outcome data were derived from medical records rather than prospectively collected, introducing the possibility of incomplete data capture, documentation variability, and information bias. Furthermore, the absence of prospective blinding of outcome assessors may have contributed to detection bias.

The non-randomized allocation of patients, based on patient preference and surgeon consensus, introduces potential selection bias. Although baseline characteristics were comparable between groups, residual confounding from unmeasured variables—including patient motivation, activity level, rehabilitation adherence, and pain perception—cannot be fully excluded. Consequently, causal inferences regarding comparative treatment effectiveness should be interpreted cautiously.

The relatively modest sample size (*n* = 35 per group), while sufficient to detect substantial within-group improvements, may have limited the statistical power to detect potential between-group differences in patient-centered responder outcomes. In addition, the low number of observed complications limited statistical power for safety analyses and precluded definitive conclusions regarding comparative safety profiles. Therefore, complication-related findings should be interpreted as descriptive rather than confirmatory evidence of similar safety between the two surgical techniques. In addition, smaller sample sizes may reduce the precision and stability of ROC-derived MCID estimates. As emphasized in methodological literature, external validation of threshold values in larger prospective multicenter cohorts remains necessary before broader clinical application. Furthermore, the high AUC values observed in the ROC analyses (0.975–0.984) should be interpreted with appropriate caution. Although these values indicate excellent discriminative performance, they may partly reflect the relatively homogeneous patient population and narrow distribution of delta scores inherent to a single-center cohort. Optimistic AUC estimates are a recognized limitation of small-sample ROC analyses, and independent external validation in larger and more heterogeneous surgical populations is necessary to confirm the robustness and generalizability of the derived MCID thresholds.

Follow-up was limited to 12 months, precluding assessment of recovery durability beyond one year and the incidence of delayed complications such as tendon elongation, persistent weakness, or late re-rupture. Additionally, the single-center, single-surgeon design, while improving procedural consistency, an unacceptable may limit generalizability to different clinical environments and varying levels of surgical expertise.

Furthermore, ROC-derived PASS threshold estimation was not performed in the present study, as the limited number of patients reporting a non-acceptable symptom state (*n* = 10) would have yielded statistically unstable cut-off values. Accordingly, PASS was operationalized exclusively as a patient-reported achievement rate using a dichotomous anchor question, consistent with the original methodology described by Tubach et al. Future studies with larger cohorts and a more balanced distribution of PASS responses are warranted to establish population-specific PASS thresholds for surgically treated Achilles tendon rupture patients. In particular, intentional oversampling of non-responders may facilitate more robust ROC-based PASS threshold estimation and improve the precision of future patient-centered outcome analyses.

Another important limitation is the absence of objective biomechanical and performance-based functional assessments, including isokinetic strength testing, heel-rise endurance, and imaging-based evaluation of tendon elongation. Such measures would provide a more comprehensive characterization of tendon healing and functional restoration beyond patient-reported outcomes alone.

Finally, no formal adjustment for multiple comparisons was performed, which may increase the risk of type I error. However, the consistency of findings across multiple independent outcome domains—including pain, functional recovery, MCID achievement, PASS rates, and GROC-defined clinical success—supports the overall robustness and internal consistency of the results. Nevertheless, the findings should be interpreted within the methodological constraints inherent to retrospective comparative research. Taken together, these limitations restrict causal inference and preclude definitive conclusions regarding the clinical comparability of the two techniques; therefore, the results should be considered hypothesis-generating and require confirmation in prospective, adequately powered studies.

The findings of the present study carry several important implications for clinical practice and future research. Both minimally invasive surgery (MIS) and open repair demonstrated substantial and clinically meaningful recovery at 12 months, with no significant differences observed across conventional outcome measures or patient-centered responder analyses. These findings suggest that both techniques represent effective treatment strategies for acute Achilles tendon rupture when performed using standardized surgical principles and postoperative rehabilitation protocols.

From a rehabilitation perspective, restoration of normal gait mechanics is one of the principal objectives following Achilles tendon repair. Although the present study focused on patient-reported outcomes and clinically meaningful improvement thresholds, the observed improvements in ATRS, AOFAS, and Thermann scores likely reflect progressive recovery of walking ability, push-off strength, and overall functional mobility during the postoperative rehabilitation period. Contemporary rehabilitation protocols emphasize early mobilization, progressive weight-bearing, and task-specific gait retraining to facilitate tendon remodeling while minimizing muscle atrophy and gait asymmetry [34,35]. Previous rehabilitation research has highlighted that gait recovery represents a critical component of functional restoration and should be considered alongside traditional patient-reported outcome measures when evaluating treatment success [36]. Furthermore, emerging technology-assisted gait rehabilitation systems may provide additional opportunities to optimize functional recovery and monitor gait performance during rehabilitation following lower-extremity injuries [37]. Therefore, the high rates of MCID and PASS achievement observed in both treatment groups may not only reflect pain reduction and functional improvement but also successful reintegration into daily activities and restoration of efficient locomotor function. Future studies incorporating objective gait analysis, wearable sensors, or instrumented walking assessments may provide additional insight into the relationship between patient-reported outcomes and biomechanical recovery following Achilles tendon repair.

An important contribution of the present study is the integration of responder-based outcome measures, including MCID, PASS, and GROC analyses, into the evaluation of surgical effectiveness. Although mean improvements between groups showed no statistically significant differences, the incorporation of patient-centered thresholds provided a more clinically interpretable assessment of recovery by determining whether postoperative improvement was meaningful from the patient perspective. The consistency of findings across both conventional and responder-based analytical frameworks suggests that treatment comparability extends beyond statistical averages to the level of individual patient-perceived recovery and satisfaction.

The MCID thresholds and PASS rates of the current study provide preliminary, population-specific estimates that may serve as context-specific references for future research; however, they should not be interpreted as definitive benchmarks for broader clinical application without external validation. Existing thresholds in the literature remain heterogeneous and are frequently derived from mixed operative and non-operative cohorts. The current findings emphasize the importance of population-specific anchor-based analyses when interpreting treatment success and patient satisfaction in surgical studies. It is important to emphasize that these MCID estimates were derived from a single-center cohort with a modest sample size and have not undergone external validation; therefore, they should be regarded as exploratory and require confirmation in larger, independent populations before broader clinical application.

From a clinical perspective, the present findings suggest that surgical decision-making between MIS and open repair should not rely solely on assumptions of superior patient-centered outcomes associated with minimally invasive approaches. Instead, factors such as surgeon experience, soft tissue condition, wound risk profile, patient preference, technical familiarity, and rehabilitation strategy may play a more important role in determining the optimal surgical approach for individual patients. Given the comparable patient-centered outcomes observed at 12 months, both techniques appear capable of achieving similarly meaningful recovery when appropriately indicated and carefully performed. Standardized rehabilitation protocols should continue to be considered an important determinant of postoperative recovery in comparative Achilles tendon research.

Future research should prioritize prospective multicenter studies with larger sample sizes and longer follow-up durations to validate the MCID thresholds and responder-based findings identified in the present study. Extended follow-up beyond one year is necessary to determine the durability of recovery and evaluate delayed complications such as tendon elongation, persistent weakness, and late re-rupture. In addition, incorporation of objective biomechanical and performance-based assessments—including isokinetic plantarflexion strength, heel-rise endurance, gait analysis, and imaging-based evaluation of tendon elongation—would provide a more comprehensive understanding of postoperative recovery beyond patient-reported outcomes alone.

Further investigation into the biological and biomechanical mechanisms underlying Achilles tendon healing also remains warranted. Studies integrating vascular imaging, tendon morphology assessment, adhesion quantification, and inflammatory biomarker profiling may further clarify the mechanistic differences between surgical approaches and their relationship to clinical recovery. Finally, future research should focus on identifying patient-level predictors of clinically meaningful recovery, which may facilitate more individualized and patient-centered treatment strategies rather than relying solely on group-level comparisons.

## 5. Conclusions

The present study indicates that both minimally invasive internal splinting and open Achilles tendon repair were associated with substantial and clinically meaningful recovery at 12 months. No statistically significant differences were observed between techniques in functional outcomes, MCID achievement rates, PASS rates, or GROC-defined clinical success within the present cohort. Although MIS may offer theoretical biological and perioperative advantages, these did not translate into superior patient-centered outcomes during the follow-up period evaluated in this study.

The integration of anchor-based responder analyses provided additional insight into patient-perceived recovery beyond conventional mean-based outcome comparisons. Furthermore, the population-specific MCID thresholds derived in the present study should be considered preliminary and exploratory, requiring external validation in larger independent cohorts before broader clinical application.

Future prospective multicenter studies with larger sample sizes, longer follow-up durations, and external validation of patient-centered outcome thresholds are warranted to confirm and extend these findings.

## Figures and Tables

**Table 1:** Baseline Demographics and Clinical Characteristics of the Study Cohort (*n* = 70).

Demographics	MIS (*n* = 35)	Open Repair (*n* = 35)	*p*-Value	SMD
Sex (Female/Male)	14/21	14/21	1.000	0.000
Age (years)	42.26 ± 8.30	42.74 ± 8.39	0.808	0.058
BMI (kg/m^2^)	27.61 ± 2.19	27.62 ± 2.18	0.987	0.004
Smoking	8 (22.9%)	8 (22.9%)	1.000	0.000
Diabetes	3 (8.6%)	3 (8.6%)	1.000	0.000
Clinical Metrics				
Side			1.000	0.000
Right	19 (54.3%)	19 (54.3%)	1.000	0.000
Left	16 (45.7%)	16 (45.7%)	1.000	0.000
Dominant side injured			1.000	0.000
Yes	19 (54.3%)	19 (54.3%)	1.000	0.000
No	16 (45.7%)	16 (45.7%)	1.000	0.000
Mechanism of injury			1.000	0.000
Basketball	9 (25.7%)	9 (25.7%)	1.000	0.000
Fall	6 (17.1%)	6 (17.1%)	1.000	0.000
Football	7 (20.0%)	7 (20.0%)	1.000	0.000
Running	8 (22.9%)	8 (22.9%)	1.000	0.000
Other	5 (14.3%)	5 (14.3%)	1.000	0.000
Rupture level (cm)	4.71 ± 0.83	4.76 ± 0.84	0.831	0.051
Time to surgery (days)	5 (3–8)	5 (3–8)	1.000	N/A

Data are presented as *n* (%) for categorical variables, mean ± standard deviation for normally distributed continuous variables, and median (minimum–maximum) for non-normally distributed variables. Standardized mean differences (SMDs) were calculated to assess baseline comparability between groups. SMD values < 0.10 indicate negligible between-group imbalance. SMDs were calculated using pooled standard deviations for continuous variables and proportion-based formulas for categorical variables. SMD was not calculated for non-normally distributed variables presented as median (minimum–maximum) and is reported as N/A (not applicable). MIS, minimally invasive surgery; BMI, body mass index; SMD, standardized mean difference; N/A, not applicable.

**Table 2:** Intra-group comparison of preoperative and final follow-up functional scores.

	Preoperative	Final Follow-Up	*p*-Value
MIS Group (*n* = 35)			
VAS score	8.05 ± 0.29	3.16 ± 0.59	<0.001
AOFAS score	44.71 ± 2.08	83.57 ± 3.73	<0.001
ATRS	42.71 ± 2.08	81.57 ± 3.73	<0.001
Thermann score	44.71 ± 2.08	82.57 ± 3.73	<0.001
Open Repair Group (*n* = 35)			
VAS score	8.06 ± 0.29	3.23 ± 0.56	<0.001
AOFAS score	44.69 ± 2.18	83.26 ± 3.64	<0.001
ATRS	42.69 ± 2.18	81.26 ± 3.64	<0.001
Thermann score	44.69 ± 2.18	82.26 ± 3.64	<0.001

Data are presented as mean ± standard deviation for normally distributed continuous variables. VAS, visual analogue scale; AOFAS, American Orthopaedic Foot and Ankle Society score; ATRS, Achilles Tendon Total Rupture Score; MIS, minimally invasive surgery.

**Table 3:** Comparison of clinical improvement (delta scores) between MIS and Open Repair groups.

Clinical Improvements	MIS Group (*n* = 35)	Open Repair Group (*n* = 35)	*p*-Value
Delta VAS	−4.80 (−6.00 to −4.30)	−4.80 (−5.40 to −4.20)	0.731
Delta AOFAS	39 (35–44)	39 (34–41)	0.559
Delta ATRS	39 (35–44)	39 (34–41)	0.559
Delta Thermann	38 (34–43)	38 (33–40)	0.559

Data are presented as median (minimum–maximum) for non-normally distributed variables. VAS, visual analogue scale; AOFAS, American Orthopaedic Foot and Ankle Society score; ATRS, Achilles Tendon Total Rupture Score; MIS, minimally invasive surgery.

**Table 4:** ROC curve analysis results for determining the Minimal Clinically Important Difference (MCID).

Delta	AUC	95% CI	Cut-Off (MCID)	Sensitivity (%)	Specificity (%)	PPV (%)	NPV (%)	*p*-Value
VAS	0.975	0.906–0.998	<−4.8	88	95	97.8	76.0	<0.001
AOFAS	0.984	0.920–0.999	>38	84	100	100.0	71.4	<0.001
ATRS	0.984	0.920–0.999	>38	84	100	100.0	71.4	<0.001
Thermann	0.984	0.920–0.999	>37	84	100	100.0	71.4	<0.001

AUC, area under the curve; CI, confidence interval; MCID, minimal clinically important difference; PPV, positive predictive value; NPV, negative predictive value; VAS, visual analogue scale; AOFAS, American Orthopaedic Foot and Ankle Society score; ATRS, Achilles Tendon Total Rupture Score. MCID thresholds were determined using the Youden index from ROC curve analysis, with the Global Rating of Change (GROC) scale as the external anchor (positive anchor: GROC scores 4–5, *n* = 50; reference: GROC scores 1–3, *n* = 20). PPV and NPV were calculated based on sensitivity, specificity, and the responder prevalence (GROC positive rate = 50/70).

**Table 5:** Comparison of MCID achievement rates between the MIS and Open repair groups.

MCID Achievement	MIS Group (*n* = 35)	Open Repair Group (*n* = 35)	*p*-Value
VAS MCID	15 (42.9%)	15 (42.9%)	1.000
AOFAS MCID	22 (62.9%)	20 (57.1%)	0.626
ATRS MCID	22 (62.9%)	20 (57.1%)	0.626
Thermann MCID	22 (62.9%)	20 (57.1%)	0.626

Data are presented as *n* (%). MCID thresholds: VAS reduction exceeding 4.8 points; AOFAS increase >38 points; ATRS increase >38 points; Thermann increase >37 points.

**Table 6:** Comparison of patient acceptable symptom state and clinical success (GROC) rates.

	MIS (*n* = 35)	Open Repair (*n* = 35)	*p*-Value
Acceptable state			1.000
Yes	30 (85.7%)	30 (85.7%)	
No	5 (14.3%)	5 (14.3%)	
Clinical success (GROC)			1.000
Success (Improvement)	25 (71.4%)	25 (71.4%)	
Non-success (No improvement)	10 (28.6%)	10 (28.6%)	

Clinical success was defined as a GROC score of 4 or 5 on a 5-point scale. GROC: Global Rating of Change.

**Table 7:** Comparison of Postoperative Complications Between MIS and Open Repair Groups.

Complication	MIS (*n* = 35)	Open Repair (*n* = 35)	*p*-Value
Superficial infection	1 (2.9%)	2 (5.7%)	1.000
Deep infection	0 (0%)	0 (0%)	—
Re-rupture	1 (2.9%)	2 (5.7%)	1.000
Wound necrosis	0 (0%)	2 (5.7%)	0.493
Sural nerve injury	0 (0%)	0 (0%)	—

Data are presented as *n* (%). *p*-values calculated using Fisher’s exact test. Dashes indicate that statistical comparison was not applicable due to absence of events in both groups.

## Data Availability

The data presented in this study are available on request from the corresponding author.

## Abbreviations

The following abbreviations are used in this manuscript:

MIS	Minimally Invasive Surgery
MCID	Minimal Clinically Important Difference
PASS	Patient Acceptable Symptom State
VAS	Visual Analogue Scale
GROC	Global Rating of Change
AOFAS	American Orthopaedic Foot and Ankle Society
ATRS	Achilles Tendon Total Rupture Score
ROC	Receiver Operating Characteristic
AUC	Area Under the Curve
PROM	Patient-Reported Outcome Measure
